# Real-World Clinical Outcomes after Genomic Profiling of Circulating Tumor DNA in Patients with Previously Treated Advanced Non-Small Cell Lung Cancer

**DOI:** 10.3390/curroncol29070382

**Published:** 2022-07-08

**Authors:** Steven Olsen, Jiemin Liao, Hidetoshi Hayashi

**Affiliations:** 1 Department of Medical and Clinical Affairs, Guardant Health Japan Corp., Minato-ku, Tokyo 105-7590, Japan; 2 Department of Outcomes and Evidence, Guardant Health Inc., Redwood City, CA 94063, USA; jiliao@guardanthealth.com; 3 Department of Medicine, Kindai University, Osaka-Sayama, Osaka 589-8511, Japan; hidet31@med.kindai.ac.jp

**Keywords:** actionable alterations, comprehensive genomic profiling, ctDNA, non-small cell lung cancer, targeted therapy

## Abstract

Comprehensive genomic profiling for advanced non-small cell lung cancer (NSCLC) can identify patients for molecularly targeted therapies that improve clinical outcomes. We analyzed data from 3084 patients (median age 65 years, 72.9% with adenocarcinoma) with advanced NSCLC registered in a real-world healthcare claims database (GuardantINFORM^TM^, Guardant Health) who underwent next-generation sequencing (NGS)-based circulating tumor DNA (ctDNA) testing (Guardant360^®^, Guardant Health) after first-line therapy (28.0% with agents targeted against genomic alterations). ctDNA was detected in 2771 samples (89.9%), of which 41.9% harbored actionable alterations, most commonly *EGFR* (epidermal growth factor receptor) mutations (29.7%). Actionable alterations were detected in 26.7% of patients (534/2001) previously treated with non-targeted agents. Emerging potentially targetable mutations were found in 40.1% (309/770) of patients previously treated with targeted therapies. Among patients with qualifying alterations detected by ctDNA testing, the time to treatment discontinuation (median 8.8 vs. 4.2 months; hazard ratio 1.97, *p* < 0.001) and overall survival (median 36.1 vs. 16.6 months; hazard ratio 2.08, *p* < 0.001) were longer for those who received matched second-line therapy versus unmatched second-line therapy. In real-world practice, results of a blood-based NGS assay prior to second-line treatment inform therapeutic decisions that can improve clinical outcomes for patients with advanced NSCLC.

## 1. Introduction

Treatment guidelines recommend testing for molecular biomarkers in advanced (unresectable stage III or stage IV) non-small cell lung cancer (NSCLC) to guide therapeutic decisions [1,2,3,4]. Guidelines recommend the assessment of genomic alterations, including mutations in *EGFR (epidermal growth factor receptor)*, *BRAF (B-Raf proto-oncogene, serine/threonine kinase)*, *KRAS* (*KRAS proto-oncogene, GTPase*), and *ERBB2* (*erb-b2 receptor tyrosine kinase 2*); rearrangements in *ALK* (*ALK receptor tyrosine kinase*), *ROS1* (*ROS proto-oncogene 1, receptor tyrosine kinase*), *RET* (*ret proto-oncogene*), and *NTRK* (*neurotrophic receptor tyrosine kinase*); and *MET* (*MET proto-oncogene, receptor tyrosine kinase*) exon 14 skipping and amplification [3]. Knowledge of these genomic alterations should inform treatment decisions that, in turn, favorably impact clinical outcomes.

After disease progression, additional biomarker testing can help to identify potentially actionable alterations that arise as resistance mechanisms to first-line treatment or to identify driver mutations that may have been missed during initial testing. Although tumor tissue can be used as the source material for such testing, plasma-based analysis of circulating tumor DNA (ctDNA) is emerging as an alternative and is often preferred over more invasive approaches, particularly when invasive procedures might pose unacceptable risk to patients [5]. Another potential advantage of ctDNA-based analysis over tissue-based analysis is the ability of the former to detect heterogenic genomic alterations within and between metastatic sites in a single test [5].

Genomic biomarker discovery rates have been compared between ctDNA and standard tissue testing in studies of patients prior to first-line treatment of advanced NSCLC and, to a lesser extent, after disease progression [6,7,8]. The frequency of biomarker detection was generally similar, particularly for point mutations and insertions/deletions, and favorable clinical outcomes were reported when using liquid biopsy to prospectively select patients for matched therapy [7,9]. The United States (US) Food and Drug Administration (FDA) has approved two plasma-based next-generation sequencing (NGS) assays as companion diagnostics to assist with the selection of targeted therapy for patients with advanced NSCLC [10,11] (reviewed in: [5]). However, information from real-world clinical practice is limited regarding how these test results are applied and how they impact clinical outcomes, especially for patients with disease progression after initial systemic treatment of advanced NSCLC.

We sought to examine how tumor genomic profiling using ctDNA testing is applied in real-world clinical practice for patients with advanced NSCLC with disease progression after initial therapy. For this purpose, we identified such patients who underwent comprehensive NGS-based ctDNA testing (Guardant360^®^, Guardant Health) and were included in a real-world database (GuardantINFORM^TM^, Guardant Health, Redwood City, CA, USA). We assessed the assay’s ability to identify clinically relevant genomic alterations and how these findings influenced the treatment decisions and outcomes for patients with advanced NSCLC.

## 2. Materials and Methods

### 2.1. Ethics

The GuardantINFORM database is a fully deidentified database that complies with Sections 164.514 (a)–(b)1ii of the US Health Insurance Portability and Accountability Act (HIPAA) regarding the determination and documentation of statistically deidentified data. Approval from an Institutional Review Board was not required because we used deidentified patient records and there was no collection, use, or transmission of individually identifiable data.

### 2.2. Data Source and Extraction

We used secondary data from the GuardantINFORM database, which comprises anonymized genomic data for patients with advanced stage solid tumors in the US who underwent testing with a Guardant360 assay. This assay has been certified according to Clinical Laboratory Improvement Amendments and accredited by the College of American Pathologists. It has also been approved by the US FDA and New York State Department of Health for clinical testing of patients with advanced (stage III–IV) solid tumors. The assay uses hybrid capture technology and NGS to identify genomic alterations in 74 genes [12], including single-nucleotide variants and insertions/deletions in *ALK*, *BRAF*, *EGFR*, *KRAS*, *MET*, *RET*, and *ROS1*; rearrangements in *ALK*, *ROS1*, *RET*, and *NTRK1*; and *MET* amplification. Since 2018, *MET* amplification was reported only when focal; prior to that, a total *MET* copy number ≥50th percentile was classified as amplified. The assessment of microsatellite instability (MSI)-high status was added to the assay in 2018. As previously reported [12], the assay showed high sensitivity, detecting variants as low as 0.02% to 0.05% allelic fraction/2.12 copies, combined with high performance for detecting single-nucleotide variants (including for variants below the 95% limit of detection), and copy number alterations, as well as high precision for detecting variants; this accuracy was also validated using clinical samples of NSCLC, colorectal cancer, or breast cancer specimens with known *EGFR*, *KRAS*, *BRAF*, and/or *ESR1* (*estrogen receptor 1*) mutations. Furthermore, in an analysis of 10,593 samples from patients with solid tumors, the technical success rate was >99.6%), the clinical sensitivity was 85.9%, and potential actionability was 16.7% for FDA-approved on-label treatments and 72.0% for treatment/trial recommendations.

Beyond ctDNA test results, the GuardantINFORM database includes structured commercial payer claims data collected from inpatient and outpatient facilities in both academic and community settings. The claims data are provided by a commercial agreement with a data aggregator (the ‘parent dataset’), which collates high-quality industry-standard sources of anonymized patient level data in addition to 150 fully privileged payer complete datasets comprising open and closed claims. The percentage of prescription claims covered by the full parent dataset by payer type is: Commercial 78%; Medicare 0%; Medicare Advantage 16%; and Medicaid 6%. The database does not include clinical features that are not coded as claims, such as tumor biomarkers assessed using other tests or clinical response to anticancer therapy. Information on deaths is obtained from third parties and aggregated with the administrative claims data. For at least half of the deaths reported by the Centers for Disease Control, there is an encounter in the parent dataset within ≤1 month of the date of death. The GuardantINFORM database is refreshed quarterly. At the time of this study, the database included records for over 190,000 patients across 60 cancer types treated by approximately 7000 oncologists in the US.

### 2.3. Study Population

For this study, we included all NSCLC patients who underwent genomic profiling with the Guardant360 assay between 1 June 2014 and 30 September 2021. The assay is indicated for people with advanced solid tumors, including unresectable stage III or stage IV (metastatic) NSCLC. We identified patients who underwent testing within 90 days prior to initiating second-line systemic therapy and who had a 6-month clean window of claims information (≥2 medical or pharmacy claims) without treatment prior to the first observed therapy date. This clean window was used to ensure that the first observed systemic therapy date corresponds to first-line treatment.

### 2.4. Treatment Lines

Treatment regimens were reconstructed over the study period using the claims data. We included all systemic cancer therapies recommended for advanced NSCLC in the National Comprehensive Cancer Network (NCCN) guidelines [3]. If the same regimens were used within 90 days after the end of one administration period to the start of the next administration date, they were combined and considered the same treatment line. Changes to a treatment regimen, including an additional drug, that occurred more than 21 days after treatment initiation were considered a new line of therapy. Discontinuing a drug without adding a new drug to a treatment regimen was not considered a new treatment line.

### 2.5. Genomic Profiling

We defined actionable genomic alterations as those listed in the NCCN guidelines for NSCLC (version 1.2022) [3], including driver and/or resistance mutations in *ALK*, *BRAF*, *EGFR*, *ERBB2*, *KRAS*, *MET*, *RET*, and *ROS1*; rearrangements in *ALK*, *ROS1*, *RET*, and *NTRK1*; *MET* amplification; and MSI-high status. *NTRK2* and *NTRK3* fusions and tumor mutation burden status were not assessed in the Guardant360 assay used at the time of data collection. The results of programmed death-ligand 1 (PD-L1) testing and tumor mutation burden assessment were not included in the database. For patients who received non-targeted first-line therapy, qualifying alterations were defined as any actionable alterations listed by NCCN. For patients who received targeted first-line therapy, qualifying alterations were defined as previously untargeted actionable alterations or emerging resistance mutations in genes of proteins previously targeted.

Only the results of Guardant360 tests were considered for the assessment of actionable genomic profiles and matched therapy because the GuardantINFORM database does not record results of other biomarker/genomic tests. Targeted therapies prescribed to patients whose ctDNA tests did not detect an actionable alteration were classified as unmatched. Targeted therapies were considered matched if they were directed at previously untreated NSCLC driver alterations or emerging resistance alterations. Any reasonable attempt at targeting the alteration was accepted as matched therapy, regardless of the US FDA approval status of the treatment. Any therapies directed toward previously treated alterations were classified as unmatched. Immunotherapy was considered matched only for patients with tumor MSI-high status because information on tumor PD-L1 status and mutation burden was not in the database. If the first-line targeted treatment was continued into the second line without the addition of a new therapy, it was classified as unmatched, but the addition of a targeted therapy directed toward a newly detected genomic alteration classified the regimen as matched.

### 2.6. Patient Outcomes

For this study, we evaluated the time to treatment discontinuation (TTD) as a surrogate for progression-free survival. TTD was defined as the time from the first day of therapy to the estimated last day of therapy, the last claim activity date, or death, whichever occurred first. Patients were censored due to a lack of follow-up information if the last claim activity date was prior to the end of therapy or <90 days after the end of therapy. We also evaluated overall survival (OS), which was defined as time from the ctDNA report date prior to second-line therapy until death. Patients were censored at the last claim activity date.

### 2.7. Statistical Analysis

All eligible patients were included in the analysis of baseline characteristics. We calculated the means, medians, and standard deviations (SD) for continuous variables, and counts and proportions for categorical variables. Proportions were compared using the two-proportion z-test or Fisher’s exact test for small samples.

TTD and OS for anticancer therapies administered after ctDNA testing were evaluated using the Kaplan–Meier method for four subgroups of patients: (1) patients with a clinically actionable ctDNA profile who received appropriately matched therapy; (2) patients with a clinically actionable ctDNA profile who did not receive matched therapy; (3) patients without a clinically actionable ctDNA profile; and (4) patients in whom ctDNA was not detected. TTD and OS were compared across the four subgroups using log-rank tests. Cox proportional hazards model was performed with these four subgroups to obtain pairwise hazard ratios (HRs) and 95% confidence intervals (CIs). In all analyses, a *p*-value of <0.05 was considered statistically significant.

All analyses were conducted using SAS software package 9.4 (SAS Institute, Cary, NC, USA).

## 3. Results

### 3.1. Patients

A total of 71,419 patients with primary NSCLC were identified in the GuardantINFORM database. The Guardant360 test was performed within 90 days before initiating second-line therapy in 3468 patients, of whom 3355 had at least two medical or pharmacy claims in the 6-month period prior to first-line therapy. A total of 3084 patients with the diagnosis of NSCLC as indicated on their Guardant360 test requisition form at second-line treatment were included in the analyses (Figure S1). Among these, 1727 (56%) were female, and the median age was 65 years. A specific claim with at least one diagnostic code for metastatic disease was filed for 2087 patients (67.7%); the other patients had advanced NSCLC not otherwise specified. The most common histologic subtype was adenocarcinoma (2247, 72.9%) (Table 1).

Non-targeted anticancer agents were administered to 2219 patients as first-line therapy for advanced NSCLC. The median age of this group of patients was 66 years, and about half (52.4%) were female (Table 1). Non-squamous carcinoma was the most frequent histological subtype (71.3%), followed by squamous cell carcinoma (14.5%). The histological type was not specified for 14.2%.

First-line targeted therapy, either alone or in combination with other agents, was administered to 865 patients (865/3084, 28.0%). The median age of this subgroup was 64 years, and nearly two-thirds of patients were female (65.4%). The most common histological type was non-squamous carcinoma (81.8%); few patients had squamous cell carcinoma (1.7%). The histological type was not specified in 16.4% of the patients.

### 3.2. First-Line Treatments

The most common first-line systemic treatment was chemotherapy (1943, 63.0%), either alone (1447, 46.9%) or in combination with other agents (496, 16.1%) (Table 2). The most common chemotherapeutic agents administered were platinum compounds (1803, 58.5%), pemetrexed (1036, 33.6%), and taxanes (703, 22.8%). Epidermal growth factor receptor (EGFR) tyrosine kinase inhibitors (TKIs) were the most common targeted agents administered (769, 24.9%), and 586 patients (19.0%) had received immune checkpoint inhibitors (ICIs). For 76 of the 207 patients treated with a vascular endothelial growth factor receptor (VEGFR) targeting agent (in all cases, bevacizumab), there was no concurrent claim for another systemic anticancer therapy.

### 3.3. ctDNA Testing Results

ctDNA was detected in plasma from 2771 of 3084 patients (89.9%), with 1160 samples (41.9%) harboring an actionable alteration (Table 3). Actionable alterations were more common in patients previously treated with targeted therapies (626/770, 81.3%) but were also present in about one-quarter of patients who had received non-targeted first-line treatment (534/2001, 26.7%). Qualifying alterations were detected in 843 samples (30.4%).

Mutations in *EGFR* were the most common overall (824, 29.7%), largely driven by a high prevalence in patients previously treated with targeted therapy (573/770, 91.5%). For example, among 490 patients who received a first- or second-generation EGFR-TKI and for whom ctDNA was detected in plasma, more than three-quarters (381/490, 77.8%) had *EGFR* driver mutations. Qualifying treatment resistance mutations included *EGFR* T790M (235, 48.0%), *MET* amplification (35, 7.1%), and *BRAF* V600E (5, 1.0%). Concurrent *KRAS* mutations were detected in 20 of these samples (3.6%), 3 of which were *KRAS* G12C. Among 217 patients previously treated with a third-generation EGFR-TKI, ctDNA was detected in plasma from 205. Among these samples, *EGFR* driver mutations were present in 179 (87.3%); resistance mutations included *MET* amplification (16, 8.9%), *EGFR* C797S (10, 5.6%), and *BRAF* V600E (8, 4.5%). There were seven samples with concurrent *KRAS* mutations (3.4%), including one with *KRAS* G12C. Resistance mutations in *EGFR*, such as T790M and C797S, were also detected in plasma from patients for whom the database had no record of prior EGFR-directed therapies.

Other alterations more prevalent among patients previously treated with targeted therapies included *MET* amplification (6.9% vs. 2.3%), *ALK* fusion (4.9% vs. 1.3%), and *BRAF* V600X (1.9% vs. 0.9%).

*KRAS* mutations were more often detected in plasma from patients not previously treated with targeted therapy (387/2001, 19.3%) than in those who had received targeted therapy (29/770, 3.8%). Nearly one-third of all such examples were *KRAS* G12C (135/416, 32.5%). *ERBB2* mutations also occurred more frequently among patients not previously exposed to targeted therapy (2.1% vs. 0.1%).

### 3.4. Treatment Decisions after ctDNA Testing

Among all patients included in this analysis, second-line targeted therapy was administered to 928 (30.1%) and was matched to the ctDNA test results for 433 (14.0%) (Table 4). The use of targeted therapy was similar regardless of whether ctDNA was detected or not (29.9% and 31.9%, respectively). Second-line targeted therapies were more likely to be matched when given to patients who had received prior targeted therapy vs. those who had not (30.9% vs. 9.7%; *p* < 0.001) and to those with *EGFR* T790M (83.9%). Matched targeted therapies were administered to half of the patients with qualifying alterations detected in ctDNA (433/843, 51.4%). Due to limited access to effective agents during the observation period, matched targeted therapies were offered to a few patients with a qualifying mutation and *KRAS* G12C (8/135, 5.9%) or *EGFR* exon 20 insertion (1/25, 4.0%).

### 3.5. Clinical Outcomes

**Figure 1:**
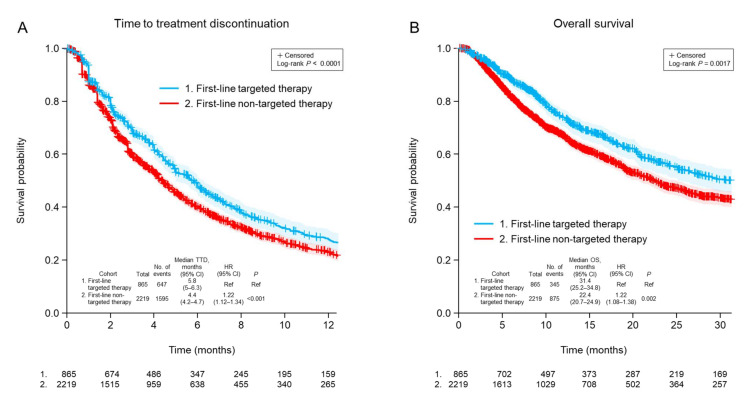
Time to discontinuation of second-line treatment (**A**) and overall survival (**B**) after ctDNA testing according to whether first-line treatment was targeted or non-targeted. CI, confidence interval; HR, hazard ratio; TTD, time to treatment discontinuation; OS, overall survival; Ref, reference.

The clinical outcomes for second-line therapy were dependent on the class of first-line therapy for advanced NSCLC. The TTD for second-line treatment was significantly longer for patients who had received targeted first-line treatment (median 5.8 months) compared with patients who had received non-targeted first-line treatment (median 4.4 months; *p* < 0.001; Figure 1A). This appeared to be driven by patients whose second-line therapy was targeted and matched, with a difference in TTD between those who received targeted or non-targeted first-line therapy (median 8.8 vs. 5.6 months; *p* = 0.006; Figure S2A). Patients with undetectable ctDNA who were therefore treated according to other clinical information had similar second-line TTD outcomes regardless of targeted or non-targeted first-line therapy (median 7.7 vs. 6.5 months *p* = 0.92; Figure S2B). A similar result was observed for OS, which was longer in patients who had received targeted first-line therapy than in those who had received non-targeted first-line therapy (median 31.4 vs. 22.4 months; *p* = 0.002; Figure 1B). However, there were no statistically significant differences in OS between any subgroups based on whether patients received first-line targeted treatment or not (Figure S3).

**Figure 2:**
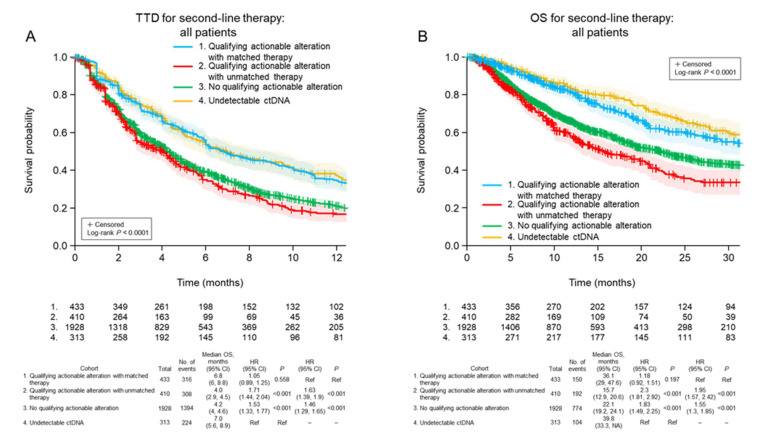
Time to discontinuation of second-line treatment (**A**) and overall survival (**B**) after ctDNA testing in the four patient cohorts, irrespective of the class of first-line treatment received. CI, confidence interval; HR, hazard ratio; TTD, time to treatment discontinuation; OS, overall survival; Ref, reference.

Clinical outcomes were then analyzed separately for the following four subgroups: (1) patients with a qualifying actionable alteration detected in ctDNA who were treated with matched targeted therapy; (2) patients with a qualifying actionable alteration detected in ctDNA who were treated with unmatched therapy; (3) patients with no qualifying actionable alteration detected in ctDNA who were treated with any therapy; and (4) patients with no detectable ctDNA who were treated with any therapy. Using these classifications, and considering all patients regardless of first-line treatment, it was found that second-line TTD was significantly longer when patients with qualifying actionable alterations detected in ctDNA were treated with matched targeted therapy rather than with unmatched therapy (median 6.8 vs. 4.0 months for matched vs. unmatched therapy; *p* < 0.001; Figure 2A). Similarly, OS was significantly longer for patients who received matched targeted therapy than for those who received unmatched therapy for qualifying actionable alterations (median 36.1 vs. 15.7 months; *p* < 0.001; Figure 2B).

### 3.6. Clinical Outcomes of Patients Who Received Non-Targeted First-Line Therapy

**Figure 3:**
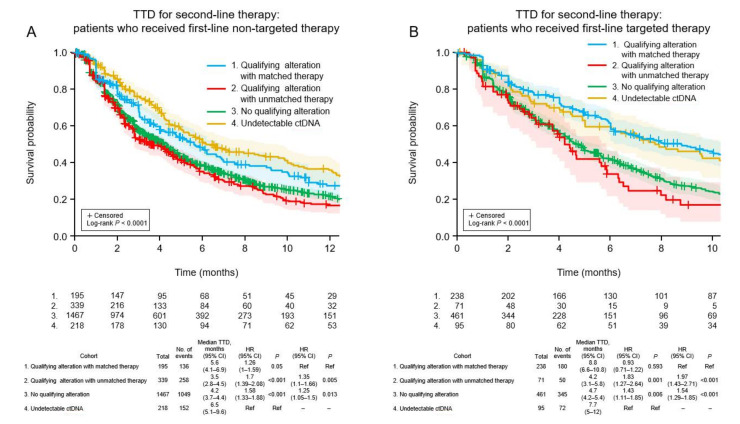
Time to discontinuation of second-line treatment in the four patient cohorts according to whether the first-line therapy was non-targeted (**A**) or targeted (**B**). CI, confidence interval; HR, hazard ratio; TTD, time to treatment discontinuation; Ref, reference.

**Figure 4:**
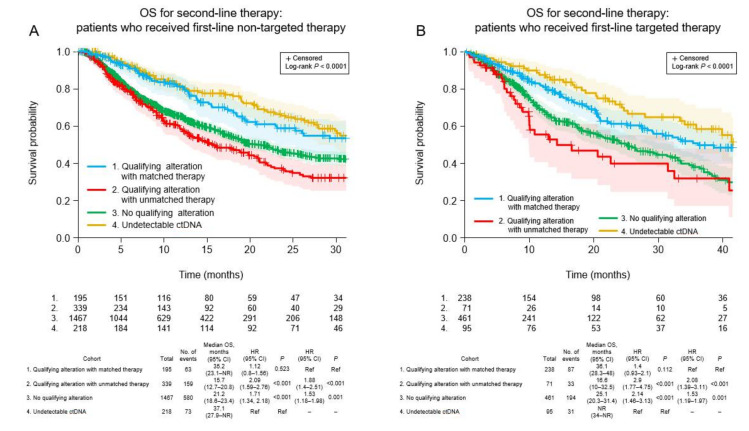
Overall survival after second-line ctDNA testing in the four patient cohorts according to whether the first-line therapy was non-targeted (**A**) or targeted (**B**). CI, confidence interval; HR, hazard ratio; NR, not reached; OS, overall survival; Ref, reference.

For second-line TTD, there was a significant trend (*p* < 0.001) among the four cohorts (Figure 3A). TTD was longest in patients for whom ctDNA could not be detected (6.5 months). The TTD was significantly longer in patients who received matched targeted therapy for actionable alterations (median 5.6 months) than patients with actionable alterations who received unmatched therapy (median 3.5 months; HR 1.35, 95% CI 1.1–1.66, *p* = 0.005) and patients in whom no actionable alterations were detected (median 4.2 months; HR 1.25, 95% CI 1.05–1.5, *p* = 0.013). A similar pattern was observed for OS (*p* < 0.001; Figure 4A), favoring patients without detectable ctDNA (median 37.1 months). Among patients with detectable ctDNA, OS was longer in patients with actionable alterations who received matched therapy (median 35.2 months) compared with patients with actionable alterations who received unmatched treatment (median 15.7 months; HR 1.88, 95% CI 1.4–2.51, *p* < 0.001) and patients in whom ctDNA was detected but did not include any actionable alterations (median 21.2 months; HR 1.53, 95% CI 1.18–1.98, *p* = 0.001).

### 3.7. Clinical Outcomes of Patients Who Received Targeted First-Line Therapy

Among patients who received targeted first-line therapy, the trend for TTD was significantly different among the four cohorts (*p* < 0.001; Figure 3B). The median TTD was similar between patients who received matched targeted therapy for new actionable alterations (8.8 months) and patients without detectable ctDNA (7.7 months; HR 0.93, 95% CI 0.71–1.22, *p* = 0.593), and was significantly longer for the former cohort compared with patients with new actionable alterations who received unmatched therapy (median 4.2 months; HR 1.97, 95% CI 1.43–2.71, *p* < 0.001) and patients in whom no new actionable alterations were detected (median 4.7 months; HR 1.54, 95% CI 1.29–1.85, *p* < 0.001). A similar pattern was observed for OS (*p* < 0.001; Figure 4B). Median OS was longer for patients who received matched targeted therapy for new actionable alterations (median 36.1 months) than in patients with new actionable alterations who received unmatched treatment (median 16.6 months; HR 2.08, 95% CI 1.39–3.11, *p* < 0.001) and patients in whom new actionable alterations were not detected (median 25.1 months; HR 1.53, 95% CI 1.19–1.97, *p* = 0.001).

## 4. Discussion

Using a real-world database, we have demonstrated that a plasma-based comprehensive genomic profiling assay can detect qualifying actionable biomarkers in patients with advanced NSCLC who have already received one line of systemic therapy. We have also shown that patients with tumors harboring qualifying actionable alterations who received a matched targeted therapy had improved clinical outcomes compared with patients who received unmatched therapies.

Following disease progression, actionable driver mutations were found in 26.7% of the patients who had not received targeted first-line therapy. These alterations included *EGFR* mutations in 12.5% and *KRAS* G12C in 6.5%. Other alterations included *MET* amplification (2.3%), *ERBB2* mutations (2.1%), *MET* exon 14 skipping (1.0%), *BRAF* V600 mutations (0.9%), and rearrangements in *ALK*, *ROS1*, or *RET* (collectively, 2.0%; 40/2001 patients). The frequencies of these alterations detected after first-line therapy for advanced NSCLC are somewhat lower than those expected for non-Asian patients with untreated advanced NSCLC based on the results of tissue testing [13] or ctDNA testing platforms [14]. A depletion in the proportion of tumors with actionable genomic alterations is expected in the second-line setting because our study population was likely enriched for patients who were tested and had no detectable tumor biomarkers prior to first-line treatment.

Among patients who received targeted therapy in the first-line setting, an actionable alteration was detected in plasma from 81.3% of all patients (626 of 770 with detectable ctDNA) and from 81.6% of those patients previously treated with EGFR-TKI (567/695). This real-world performance compares favorably to clinical trial data for positive percent agreement (sensitivity) of ctDNA-based assays in the detection of *EGFR* mutations (droplet digital polymerase chain reaction (PCR), 51–81%; NGS, 50–86%) in the plasma of patients with *EGFR*-mutated NSCLC previously identified through tissue testing [15]. Emerging resistance mutations were detected in nearly half of the patients who received first-line targeted therapy (309/626, 49.4%). This was largely but not exclusively driven by the presence of *EGFR* T790M in patients previously treated with a first- or second-generation EGFR-TKI (235/490, 48.0%), consistent with other studies of patients treated with a first- or second-generation EGFR-TKI [16,17] but higher than the detection rate of 26.8% reported for a US FDA-approved PCR-based plasma test in a similar population [18]. In patients who received a third-generation EGFR-TKI as first-line therapy, the most common emerging and potentially actionable alterations were *MET* amplification (7.8%), *EGFR* C797S (4.9%), and *BRAF* V600E (3.9%). These rates were similar to those reported in an analysis of 91 of 279 patients treated with first-line osimertinib in the FLAURA study, in which the frequencies of *MET* amplification, *EGFR* C797X, and *BRAF* V600E were 15%, 7%, and 3%, respectively [19].

Matched therapy was administered to only half of the patients with qualifying alterations (433/843, 51.4%). This was partly due to the lack of established targeted therapies for certain qualifying alterations during the observation period. The use of matched therapy was relatively low in patients with tumor alterations such as *ERBB2* mutation (14/43, 32.6%), *MET* amplification (32/99, 32.3%), *KRAS* G12C (8/135, 5.9%), and *EGFR* exon 20 insertion (1/25, 4.0%). Excluding these alterations, around 30% of the remaining patients with qualifying alterations did not receive matched therapy (163/541, 30.1%) despite the availability of highly effective targeted therapies. This is important, because when actionable alterations were detected in ctDNA and treated with matched targeted therapy, both the TTD and OS for second-line therapy were significantly improved compared with those of patients with qualifying alterations who were not treated with matched therapy (median TTD 6.8 vs. 4.0 months, HR 1.63, *p* < 0.001; median OS 36.1 vs. 15.7 months, HR 1.95, *p* < 0.001). These results were observed even though we classified matched therapy as any reasonable attempt by the treating physician to target a qualifying alteration, and included agents not approved by the US FDA for specific use in advanced NSCLC.

Our results in the second-line setting are qualitatively consistent with those from studies of advanced NSCLC patients tested and treated in the first-line setting. For example, in a survey of patients with advanced non-squamous NSCLC treated in US community practice, the median OS after first-line treatment was 31.8 months for patients who received targeted therapy compared with 12.7 months for patients who received other therapies [20]. In a Japanese study of patients with advanced NSCLC, targeted therapy was associated with longer OS in patients with actionable alterations in tumor tissue compared with patients without actionable tumor alterations (median OS not reached vs. 18.1 months; HR 0.44, *p* = 0.041) and compared with patients with actionable alterations who did not receive targeted therapy (median OS: not reached vs. 6.1 months; HR 0.14, *p* = 0.0027) [21]. In a Korean study of patients with advanced stage lung adenocarcinoma whose tumor biopsies were subjected to NGS testing, OS was significantly longer for patients treated with matched targeted therapy than for patients who received unmatched therapy (HR 2.58, *p* < 0.001) [22]. Similar results were seen in a German real-world retrospective analysis of previously untreated patients with advanced NSCLC. For patients with actionable tumor biomarkers, OS was significantly longer in patients who received targeted therapy than in patients who received chemotherapy [23]. In a retrospective study of American patients with advanced NSCLC, the clinical outcomes of first-line therapy were evaluated for 417 patients who underwent tumor biomarker testing by plasma or tissue NGS (287 received matched targeted therapy and 130 received unmatched therapy). Progression-free survival (adjusted HR 0.72, *p* = 0.022) and OS (adjusted HR 0.70, *p* = 0.035) were superior for patients who received matched treatment [14].

The absence of detectable ctDNA was associated with longer TTD and survival in this study. This phenomenon has been described for patients with advanced NSCLC treated with targeted therapies [24,25,26]. This may reflect the fact that lower-grade and smaller tumors, which are associated with better clinical outcomes, tend to shed less DNA into the bloodstream.

There are several limitations of this study. Treatment history was incomplete for some patients in the claims database. For example, systemic therapies used in clinical trials were not recorded, and this may explain the claims for some patients who received VEGF inhibitors in the absence of concurrent therapy. Furthermore, the mutation patterns for some patients suggested prior exposure to a targeted agent that was not recorded in the database. The ctDNA assay detected both an *EGFR* driver mutation and *EGFR* T790M in some patients classified as having no prior targeted therapy. Such findings suggest prior exposure to a first- or second-generation EGFR-TKI. It is possible that some patients may have experienced disease progression after study-related EGFR-TKI or relapse after adjuvant treatment with an early generation EGFR-TKI, but this history is unknown.

Another limitation is that the database does not include results of biomarker testing from other platforms. This information would be available to a patient’s physician and may have influenced treatment decisions. However, while patients who received targeted therapy based on the results of alternative testing platforms would have been included among those without actionable alterations, we still observed significant improvements in TTD and OS for matched therapy versus unmatched therapy based on the ctDNA findings alone.

Plasma-based NGS assays for ctDNA detection are dependent on tumor DNA shedding into the bloodstream, which may be limited in patients with a lower tumor burden or those with central nervous system metastases. As with other DNA-dependent assays, the sensitivity for the detection of complex genomic alterations such as rearrangements and large introns may be lower than with protein-based or RNA-based technologies. Nevertheless, the overall detection rates of clinically informative genomic biomarkers are similar with NGS ctDNA testing and standard tissue testing in several studies [6,7]. Even considering a potential limitation in the sensitivity of the ctDNA assay, a clinically relevant proportion of patients in this real-world setting harbored actionable genomic alterations (24.1% of the patients without prior targeted treatment, 35.7% of the patients previously treated with targeted therapy). The ctDNA assay used in this analysis is associated with a high positive predictive value for actionable alterations in other studies [6,9], which is corroborated by the favorable treatment outcomes observed in the current study when matched targeted therapies were administered to patients with actionable alterations detected by ctDNA testing. Another consideration with ctDNA assays is the unintended detection of non-tumor mutations that occur through clonal hematopoiesis of indeterminate potential (CHIP) [27]. However, actionable NSCLC driver mutations included in the NCCN guidelines are not among the common alterations associated with CHIP [27].

The selection bias for the types of patients who undergo tumor genomic testing after first-line treatment is reflected in the database. The population was enriched for patients previously treated with targeted therapy, particularly EGFR-TKI, because of the high probability of the presence of resistance mutations, such as *EGFR* T790M, *MET* amplification, and *BRAF* V600E. Although complete test history is lacking, patients who had not received prior targeted therapy potentially did not undergo complete tumor genomic testing before initial therapy for advanced disease. Patients previously tested and found to have actionable tumor biomarkers other than *EGFR* mutations were under-represented in the cohort; therefore, the potential benefit of biomarker testing after such treatments could not be clearly established.

The treatment decisions for advanced NSCLC should follow a patient-centric, holistic approach. In addition to the results of biomarker testing, factors that were not included in the database may have informed the choice of therapy. Such factors include the patient’s performance status and the presence of co-morbidities, along with personal treatment preferences and access to specific medicines.

In conclusion, to the best of our knowledge, this is the first report to describe the real-world clinical outcomes of patients who underwent plasma NGS testing immediately prior to second-line treatment for advanced NSCLC. The assay used in this study identified a significant proportion of patients as potential candidates for targeted therapy. The application of matched targeted therapy based on the ctDNA results was associated with improved TTD and OS. Our findings add to the growing body of evidence supporting the role of tumor biomarker testing, specifically plasma-based NGS, for patients with advanced NSCLC with disease progression after first-line systemic treatment.

## Figures and Tables

**Table 1:** Patient characteristics.

	All Patients	Non-Targeted First-Line Therapy	Targeted First-Line Therapy	*p*-Value ^a^
*n*	3084	2219	865	
Age (median)	65	66	64	
Female, *n* (%)	1727 (56.0%)	1162 (52.4%)	566 (65.4%)	<0.001
Histology, *n* (%)				
Non-squamous	2291 (74.3%)	1583 (71.3%)	708 (81.8%)	<0.001
Squamous	337 (10.9%)	322 (14.5%)	15 (1.7%)	<0.001
Not specified	456 (14.8%)	314 (14.2%)	142 (16.4%)	0.125

^a^*p*-values for non-targeted vs. targeted therapy.

**Table 2:** First-line treatments.

	*n* (%)
*n*	3084
Chemotherapy ± other agents	1943 (63.0%)
Immune checkpoint inhibitor ± other agents	586 (19.0%)
VEGF inhibitor ± other agents	207 (6.7%)
Targeted therapy ± other agents	865 (28.0%)
First-/second-generation EGFR-TKI	552 (17.9%)
Third-generation EGFR-TKI	217 (7.0%)
EGFR monoclonal antibody	7 (0.2%)
Other TKI	89 (2.9%)

Some patients received multiple classes of therapy; VEGF, vascular endothelial growth factor; EGFR, epidermal growth factor receptor; TKI, tyrosine kinase inhibitor.

**Table 3:** Molecular alterations identified using the Guardant360 assay in patients with detectable ctDNA.

Alteration	All Patients	Non-Targeted First-Line Treatment		Targeted First-Line Treatment		***p*-Value (Non-Targeted vs. Targeted)**
		Any Alteration	Qualifying Alteration	Any Alteration	Qualifying Alteration	
*n*	2771	2001		770		
Actionable alteration ^a^, *n* (%)	1160 (41.9%)	534 (26.7%)	534 (26.7%)	626 (81.3%)	309 (40.1%)	<0.001
*KRAS* mutation, *n* (%)	416 (15.0%)	387 (19.3%)	131 ^b^ (6.5%)	29 (3.8%)	4 ^b^ (0.5%)	<0.001
*EGFR* mutation, *n* (%)	824 (29.7%)	251 (12.5%)	251 (12.5%)	573 (77.4%)	251 (32.6%)	<0.001
Exon 19 deletion, *n* (%)	456 (16.5%)	131 (6.5%)	131 (6.5%)	325 (42.2%)	1 (0.1%)	<0.001
L858R, *n* (%)	272 (9.8%)	70 (3.5%)	70 (3.5%)	202 (26.2%)	0	<0.001
T790M, *n* (%)	292 (10.5%)	41 ^c^ (2.0%)	1 ^c^ (<0.1%)	251 (32.6%)	243 (31.6%)	<0.001
C797S, *n* (%)	24 (0.9%)	8 (0.4%)	0	16 (2.1%)	16 (2.1%)	<0.001
Other point mutation, *n* (%)	62 (2.2%)	25 (1.2%)	25 (1.2%)	37 (4.8%)	0	<0.001
Exon 20 insertion, *n* (%)	32 (1.2%)	25 (1.2%)	25 (1.2%)	7 (0.9%)	0	0.581
*MET* alterations, *n* (%)	126 (4.5%)	68 (3.4%)	67 (3.3%)	58 (7.5%)	54 (7.0%)	<0.001
Amplification, *n* (%)	99 (3.6%)	46 (2.3%)	46 (2.3%)	53 (6.9%)	53 (6.9%)	<0.001
Exon 14 skipping, *n* (%)	26 (0.9%)	21 (1.0%)	21 (1.0%)	5 (0.6%)	0	0.448
Point mutation, *n* (%)	3 (0.1%)	1 (<0.1%)	0	2 (0.3%)	2 (0.3%)	0.189
*ALK* alterations, *n* (%)	66 (2.4%)	28 (1.4%)	27 (1.3%)	38 (4.9%)	4 (0.5%)	<0.001
Fusion, *n* (%)	65 (2.3%)	27 (1.3%)	27 (1.3%)	38 (4.9%)	3 (0.4%)	<0.001
Point mutation, *n* (%)	7 (0.3%)	3 (0.1%)	0	4 (0.5%)	4 (0.5%)	0.099
*ERBB2* mutation, *n* (%)	43 (1.6%)	42 (2.1%)	42 (2.1%)	1 (0.1%)	1 (0.1%)	<0.001
*BRAF* V600X ^d^, *n* (%)	34 ^d^ (1.2%)	19 d (0.9%)	19 d (0.9%)	15 ^e^ (1.9%)	14 ^e^ (1.8%)	0.052
*RET* fusion, *n* (%)	15 (0.5%)	9 (0.4%)	9 (0.4%)	6 (0.8%)	6 (0.8%)	0.384
*ROS1* fusion, *n* (%)	10 (0.4%)	4 (0.2%)	4 (0.2%)	6 (0.8%)	0	0.033
*NTRK1* fusion, *n* (%)	2 (0.1%)	0	0	2 (0.3%)	2 (0.3%)	0.077
MSI-high, *n* (%)	3 (0.1%)	3 (0.1%)	3 (0.1%)	0	0	0.565

Some patients had multiple alterations; ^a^ According to National Comprehensive Cancer Network guidelines; ^b^ *KRAS* G12C; ^c^ Includes one case without a concurrent *EGFR* driver mutation; ^d^ All V600E except for one patient with V600K; ^e^ All V600E. *ALK*, *ALK receptor tyrosine kinase*; *BRAF*, *B-Raf proto-oncogene, serine/threonine kinase*; ctDNA, circulating tumor DNA; *EGFR*, *epidermal growth factor receptor*; *ERBB2*, *erb-b2 receptor tyrosine kinase 2*; *KRAS*, *KRAS proto-oncogene, GTPase*; *MET*, *MET proto-oncogene, receptor tyrosine kinase*; MSI, microsatellite instability; *NTRK*, *neurotrophic receptor tyrosine kinase*; *RET*, *ret proto-oncogene*; *ROS1*, *ROS proto-oncogene 1, receptor tyrosine kinase*.

**Table 4:** Second-line treatments administered.

	*n*	Targeted Therapy		Non-Targeted Therapy	
		Any, *n* (%)	Matched, *n* (%)	with ICI, *n* (%)	without ICI, *n* (%)
All patients	3084	928 ^a^ (30.1%)	433 ^a^ (14.0%)	1315 ^b^ (42.6%)	841 (27.3%)
No ctDNA detected	313	100 (31.9%)	NA	145 (46.3%)	68 (21.7%)
ctDNA detected	2771	828 ^a^ (29.9%)	433 ^a^ (15.6%)	1170 ^b^ (42.2%)	773 (27.9%)
First-line non-targeted therapy	2001	293 ^a^ (14.6%)	195 ^a^ (9.7%)	1084 ^b^ (54.2%)	624 (31.2%)
First-line targeted therapy	770	535 (69.5%)	238 (30.9%)	86 (11.2%)	149 (19.4%)
No qualifying alteration	1928	365 (18.9%)	NA	963 (49.9%)	600 (31.1%)
Any qualifying alteration	843	463 ^a^ (54.9%)	433 ^a^ (51.4%)	207 ^b^ (24.6%)	173 (20.5%)
*EGFR* driver mutation ^c^	511	373 (73.0%)	348 ^d^ (68.1%)	50 (9.8%)	88 (17.2%)
*EGFR* T790M ^c^	285	251 (88.1%)	239 ^d^ (83.9%)	10 (3.5%)	24 (8.4%)
*EGFR* exon 20 insertion	25	1 (4.0%)	1 (4.0%)	11 (44.0%)	13 (52.0%)
*KRAS* G12C ^c^	135	11 (8.1%)	8 ^d^ (5.9%)	91 (67.4%)	33 (24.4%)
*MET* amplificationc	99	41 (41.4%)	32 ^d^ (32.3%)	32 (32.3%)	26 (26.3%)
*ERBB2* mutation	43	14 (32.6%)	14 (32.6%)	15 (34.9%)	14 (32.6%)
*ALK* fusion ^c^	36	24 (66.7%)	22 d (61.1%)	4 (11.1%)	8 (22.2%)
*BRAF* V600X ^c^	33	19 (57.6%)	17 (51.5%)	9 (27.3%)	5 (15.2%)
*MET* exon 14 skipping ^c^	23	19 (82.6%)	19 (82.6%)	3 (13.0%)	1 (4.3%)
Other alteration ^c,e^	30	16 (53.3%)	14 ^a,d^ (46.7%)	7 ^b^ (23.3%)	6 (20.0%)

Some plasma samples had multiple alterations. ^a^ Includes 2 patients with MSI-high treated with ICI; ^b^ Excludes 2 patients with MSI-high treated with ICI; ^c^ Alteration present but may co-exist with the primary qualifying alteration; ^d^ In some cases, treatment may have been matched to a co-existing alteration rather than the alteration shown; ^e^ *ALK* point mutation (8), *MET* point mutation (2), *NTRK1* fusion (2), *RET* fusion (15), *ROS1* fusion (4), MSI-high (3). *ALK*, *ALK receptor tyrosine kinase*; *BRAF*, *B-Raf proto-oncogene, serine/threonine kinase*; ctDNA, circulating tumor DNA; *EGFR*, *epidermal growth factor receptor*; *ERBB2*, *erb-b2 receptor tyrosine kinase 2*; ICI, immune checkpoint inhibitor; *KRAS*, *KRAS proto-oncogene, GTPase*; *MET*, *MET proto-oncogene, receptor tyrosine kinase*; MSI, microsatellite instability; NA, not applicable; *NTRK*, *neurotrophic receptor tyrosine kinase*; *RET*, *ret proto-oncogene*; *ROS1*, *ROS proto-oncogene 1, receptor tyrosine kinase*.

## Data Availability

The datasets generated during and/or analyzed during the current study are not publicly available and cannot be shared due to the use of a third-party healthcare claims database. Researchers interested in replicating our study or pursuing new research topics should contact Guardant Health directly (https://guardanthealth.com/products/biopharma-solutions/real-world-evidence/, accessed on 5 June 2022).

## Supplementary Materials

The following supporting information can be downloaded at: https://www.mdpi.com/article/10.3390/curroncol29070382/s1, Figure S1: Disposition of patients; Figure S2: Time to discontinuation of second-line treatment according to whether first-line treatment was targeted or non-targeted in (A) patients whose second-line treatment was targeted and matched to actionable alterations and (B) patients without detectable ctDNA; Figure S3: Overall survival after ctDNA testing according to whether first-line treatment was targeted or non-targeted in (A) patients whose second-line treatment was targeted and matched to actionable alterations and (B) patients without detectable ctDNA.
