# Supplementary material for: Real-World Clinical Outcomes after Genomic Profiling of Circulating Tumor DNA in Patients with Previously Treated Advanced Non-Small Cell Lung Cancer
Source: Curr Oncol. 2022 Jul 8;29(7):4811–26. doi: 10.3390/curroncol29070382 (PMC9318660; doi:10.3390/curroncol29070382)
Supplement: Supplementary file 1 [file curroncol-29-00382-s001.zip › curroncol-1782269-supplementary.pdf]

Current Oncology

Supplementary Materials for Real-World Clinical Outcomes after Genomic Profiling of Circulating Tumor DNA in Patients with Previously Treated Advanced Non-Small Cell Lung Cancer

Steven Olsen, Jiemin Liao and Hidetoshi Hayashi

Figure S1. Disposition of patients.

Figure S2. Time to discontinuation of second-line treatment according to whether first-line treatment was targeted or non-targeted in (A) patients whose second-line treatment was targeted and matched to actionable alterations and (B) patients without detectable ctDNA;.

Figure S3. Overall survival after ctDNA testing according to whether first-line treatment was targeted or non-targeted in (A) patients whose second-line treatment was targeted and matched to actionable alterations and (B) patients without detectable ctDNA.

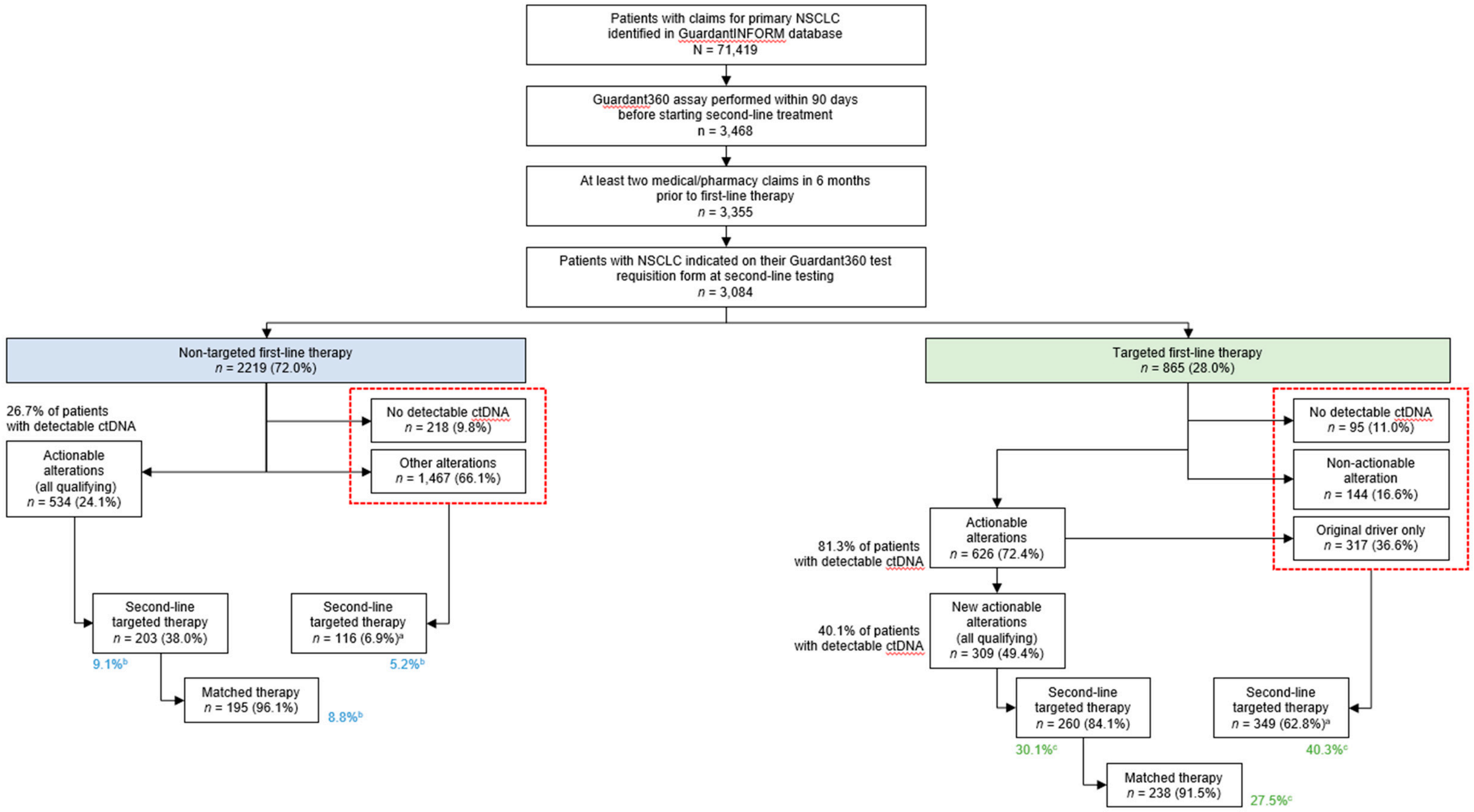

Figure S1. Disposition of patients. Actionable alterations were defined according to National Comprehensive Cancer Network guidelines as described in the text. Percentages were calculated using the value in the preceding box as the denominator unless stated otherwise. The denominator was the sum of patients in the red dashed box; [b]Denominator = 2,219 (patients who received non-targeted first-line therapy); [c]Denominator = 865 (patients who received targeted first-line therapy).

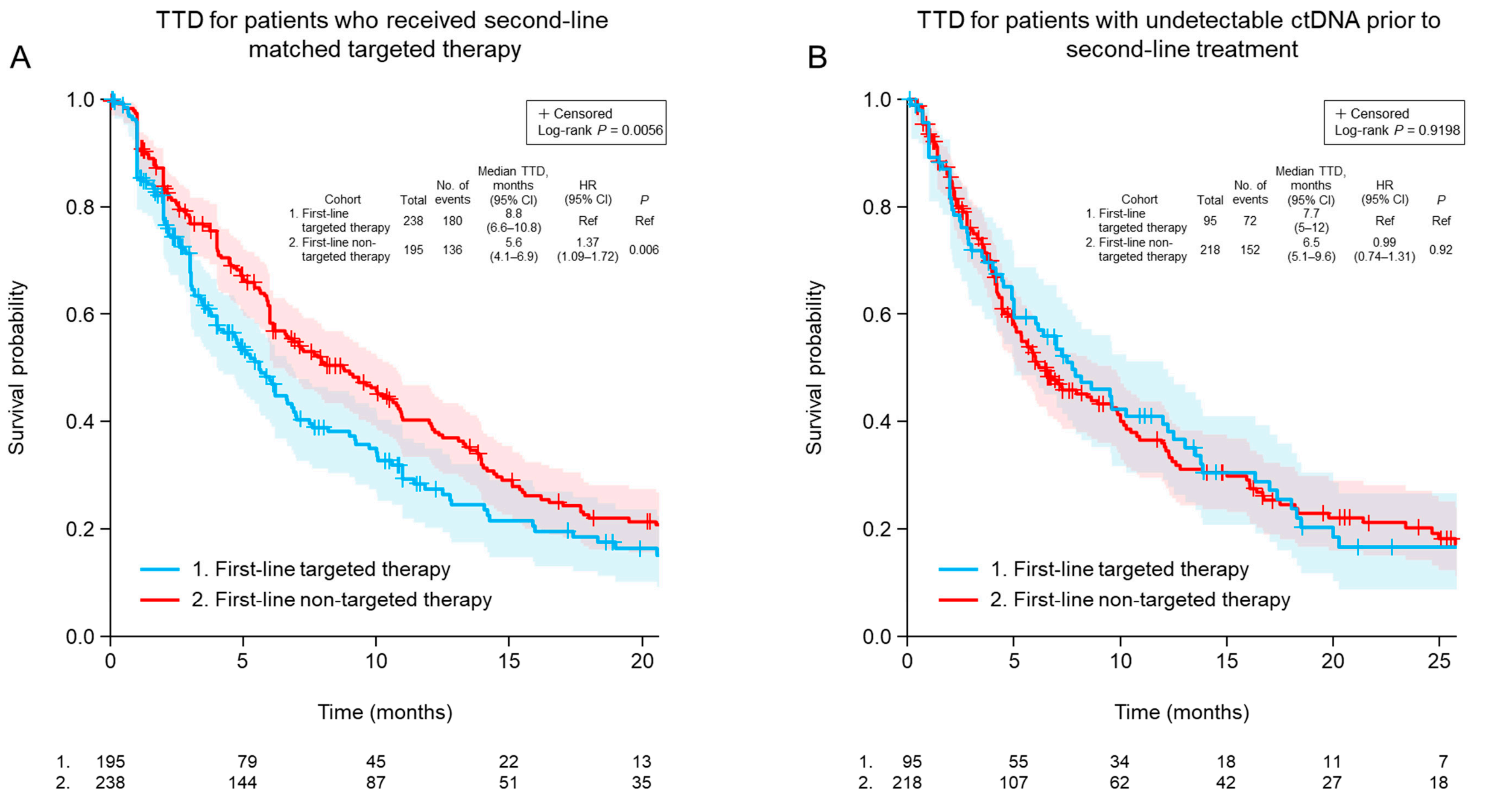

Figure S2. Time to discontinuation of second-line treatment according to whether first-line treatment was targeted or non-targeted in (A) patients whose second-line treatment was targeted and matched to actionable alterations and (B) patients without detectable ctDNA. CI, confidence interval; HR, hazard ratio; TTD, time to treatment discontinuation, Ref, reference.

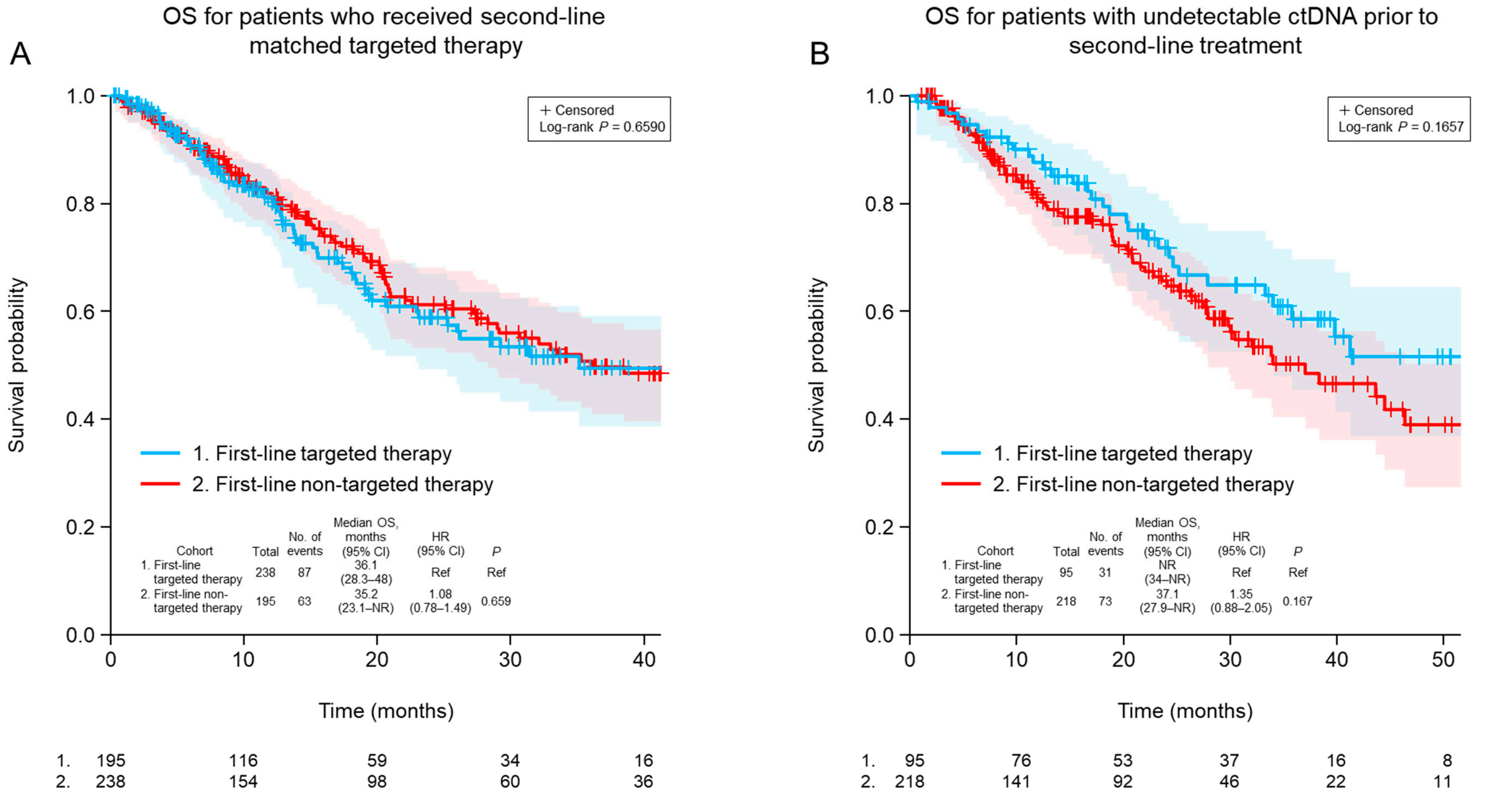

Figure S3. Overall survival after ctDNA testing according to whether first-line treatment was targeted or non-targeted in (A) patients whose second-line treatment was targeted and matched to actionable alterations and (B) patients without detectable ctDNA. CI, confidence interval; HR, hazard ratio; NR, not reached; OS, overall survival; Ref, reference.
